# Bilateral Endoscopic Endonasal Optic Nerve Decompression in an Infant with Osteopetrosis: A Case Report

**DOI:** 10.1055/a-2554-2426

**Published:** 2025-04-11

**Authors:** Rita M. Jalkh, Yara Yammine, Nader Zalaquett, Houssein Darwish, Zeina Korban

**Affiliations:** 1 Department of Otolaryngology—Head and Neck Surgery, American University of Beirut Medical Center, Beirut, Lebanon; 2 Department of Neurosurgery, American University of Beirut Medical Center, Beirut, Lebanon

**Keywords:** osteopetrosis, optic nerve decompression, endoscopic endonasal approach, pediatric, case report

## Abstract

**Background:**

Osteopetrosis is a rare genetic disorder characterized by abnormal bone density and structure, often leading to vision loss due to optic canal stenosis and consequent nerve compression. Early intervention is critical to prevent irreversible damage. This case report discusses the management of bilateral optic nerve compression in an infant with osteopetrosis.

**Case Description:**

A 7-month-old male with a family history of osteopetrosis presented with hepatosplenomegaly. The infant was diagnosed with osteopetrosis based on radiological findings and genetic testing. Ophthalmologic examination and magnetic resonance imaging showed evidence of bilateral optic nerve compression. Endoscopic transcaruncular optic nerve decompression was not attainable The patient underwent a bilateral expanded endoscopic endonasal medial orbital wall and optic canal decompression.

**Conclusion:**

This is one of the few reported cases of endoscopic endonasal optic nerve decompression surgery on an infant. Endoscopic endonasal optic nerve decompression surgery is a viable and effective treatment option for optic nerve compression in infants with osteopetrosis, especially in cases where cost of surgery is a limiting factor for patients. This approach provides direct access to the optic canal with minimal morbidity, offering significant potential for visual recovery, and an improved quality of life. Our patient represents the youngest reported infant in the literature, demonstrating the potential for undergoing this surgical approach at the earliest possible age to aid with his prognosis.

## Introduction

Osteopetrosis is a rare hereditary disorder characterized by increased bone density due to defective osteoclast function. 1 Osteoclasts are responsible for bone resorption, a critical process in bone remodeling. The failure of osteoclasts to resorb bone leads to the accumulation of dense but brittle bone, resulting in the characteristic features of osteopetrosis.

Osteopetrosis can be inherited in autosomal recessive, dominant, or X-linked forms, with the most severe cases typically autosomal recessive, caused by mutations in genes like TCIRG1, CLCN7, and OSTM1. 2 Symptoms vary by severity and include bone fragility, anemia, hepatosplenomegaly, and cranial nerve compression. 3 The latter can result in complications such as vision and hearing loss due to the narrowing of cranial foramina and subsequent nerve entrapment. 4

Early diagnosis and intervention are crucial in managing osteopetrosis, especially to prevent irreversible complications such as vision loss. Treatment can include medical management, supportive therapies, and surgery to alleviate symptoms and prevent progression. 5 Hematopoietic stem cell transplantation can be curative for certain forms of osteopetrosis, especially when performed early in life. 1 In cases where optic nerve compression is present, surgical decompression may be necessary and urgent to prevent permanent vision loss. 4

This case report aims to highlight the management of optic nerve compression in an infant with osteopetrosis through a binostril endoscopic endonasal optic nerve decompression, underscoring the importance of early diagnosis and intervention and demonstrating the viability and effectiveness of this minimally invasive surgical approach in infants, particularly in resource-limited settings.

## Management and Surgical Procedure

### Case Presentation

The patient, a 7-month-old male, initially presented to the pediatrician on March 28, 2024 for hepatosplenomegaly with evidence of leukocytosis and thrombocytopenia on laboratory tests. On physical examination, he exhibited limited limb movements, mildly tense anterior fontanelle, audible snoring, and protruding upper gingiva with poor weight gain suggestive of failure to thrive. The lungs were clear, and heart sounds were normal; however, palpable hepatosplenomegaly was present. Given the patient's family history of osteopetrosis—his parents are first-degree cousins with a known CLCN7 gene mutation—a chest X-ray was performed, revealing diffuse thick bony sclerosis of the axial and appendicular skeleton, consistent with osteopetrosis.

**Fig. 1:**
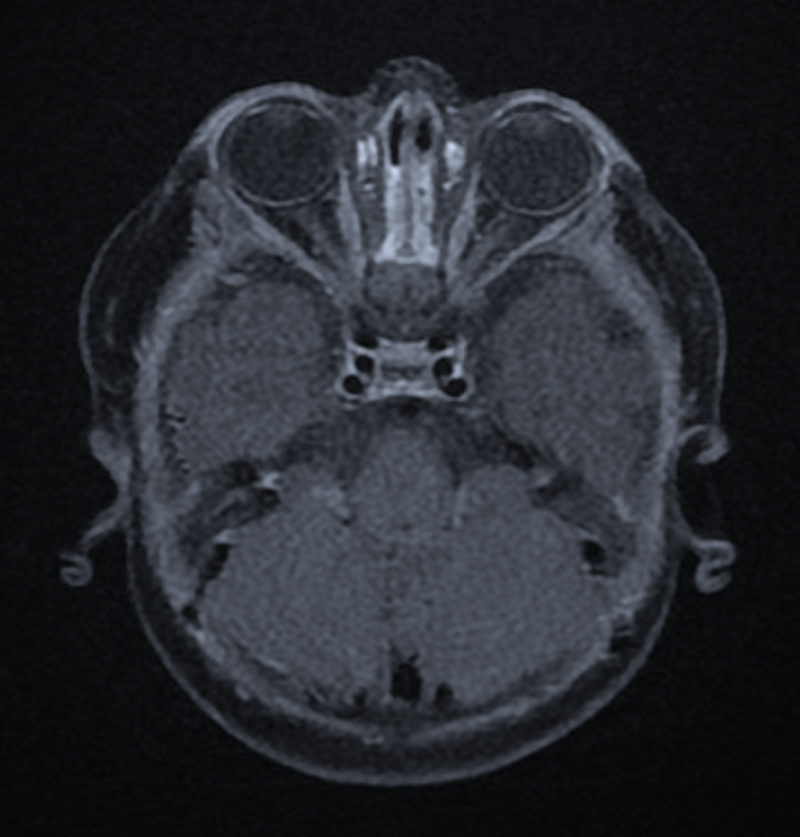
Magnetic resonance imaging axial T1-weighted: narrowed optic canals.

On April 1, 2024, an initial ophthalmologic examination revealed temporal pallor on fundoscopy without signs of optic atrophy. By April 29, 2024, magnetic resonance imaging (MRI) results confirmed small optic canals, suggesting bilateral optic nerve compression ( Fig. 1 ). Genetic testing identified a homozygous variant of uncertain significance in the CLCN7 gene, aligning with suggesting a possible diagnosis of autosomal recessive osteopetrosis type 4.

By July 1, 2024, the patient began showing a preference for downgaze, along with esotropia and pale optic nerves bilaterally, indicating worsening compressive osteopetrosis. Given the progressive nature of the disease and the increasing risk of optic nerve damage, the medical team decided to proceed with optic nerve decompression prior to bone marrow transplantation (BMT) to avoid the higher surgical mortality associated with post-BMT immunosuppression. Due to the patient's young age and small anatomical structures, the ophthalmology team opted against the transcaruncular approach. On July 25, 2024, the patient underwent successful expanded endoscopic endonasal medial orbital wall and optic canal decompression bilaterally.

### Intraoperative Course and Surgical Technique

The surgical team, comprising of rhinology, endoscopic skull base, and neurosurgery specialists, navigated the intricate anatomical structures with precision, achieving the desired decompression without intraoperative complications such as bleeding, cerebrospinal fluid (CSF) leaks and optic nerve injury. Navigation was not utilized during the procedure due to financial constraints expressed by the patient's family, yet the surgery was completed with high precision.

The patient was placed under general anesthesia and positioned supine, with the head supported on a donut headrest and a shoulder roll beneath the shoulders. Following sterile preparation of the face and nasal cavity, a 30-degree, 2.7-mm endoscope was used to access the nasal cavity. The nasal septum was infiltrated with a solution of 1% xylocaine with 1:100,000 epinephrine to facilitate dissection and reduce bleeding. A left Killian incision was made, and bilateral mucoperichondrial flaps were elevated. A portion of the cartilaginous septum was excised, followed by a posterior septectomy using a microdebrider.

**Fig. 2:**
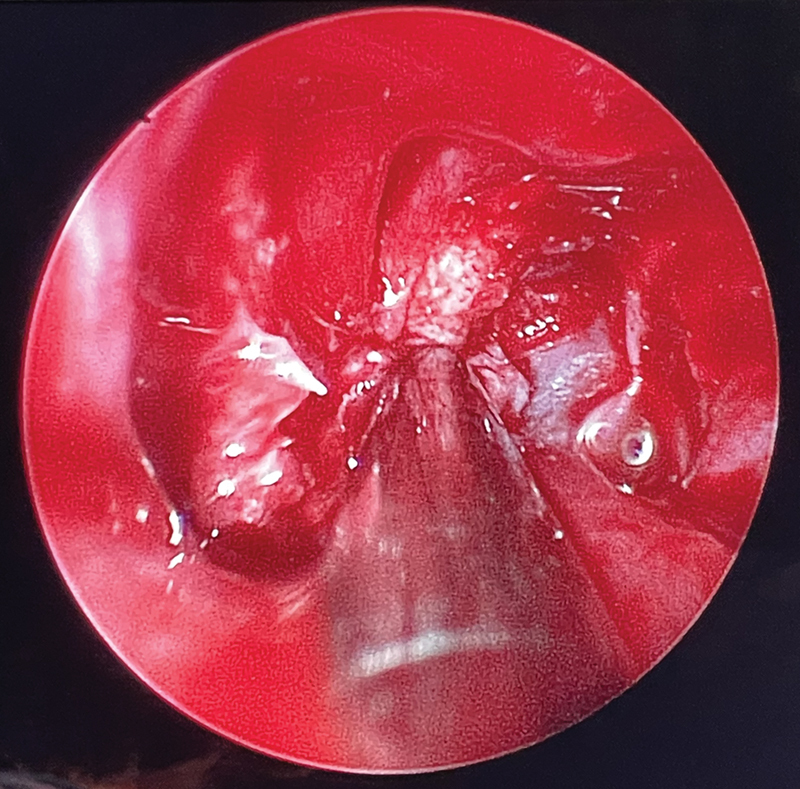
Left medial periorbita exposed (suction at the annulus of Zinn).

**Fig. 3:**
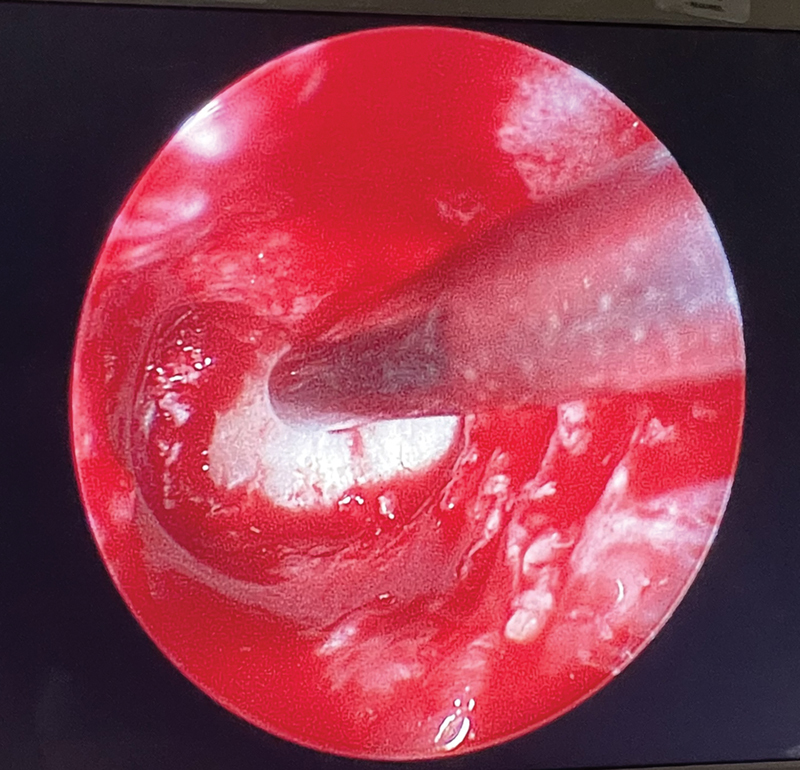
Left medial periorbita exposed.

To improve exposure and allow for precise instrumentation, the anterior one-fourth of the middle turbinate was resected bilaterally. The bone of the middle turbinate was notably ossified, necessitating careful drilling and removal with a bone curette. This was followed by a bilateral total ethmoidectomy. This approach allowed access to the medial orbital walls bilaterally. The bony walls were meticulously removed exposing the periorbita to decompress the orbits, extending the decompression posteriorly to the annulus of Zinn ( Figs. 2 , 3 ). Complete decompression of the optic canals was achieved bilaterally. Hemostasis was ensured using FloSeal, Surgicel, and Gelfoam. The patient was then transferred to the pediatric intensive care unit (PICU) while remaining intubated for close postoperative monitoring.

### Postoperative Course

**Fig. 4:**
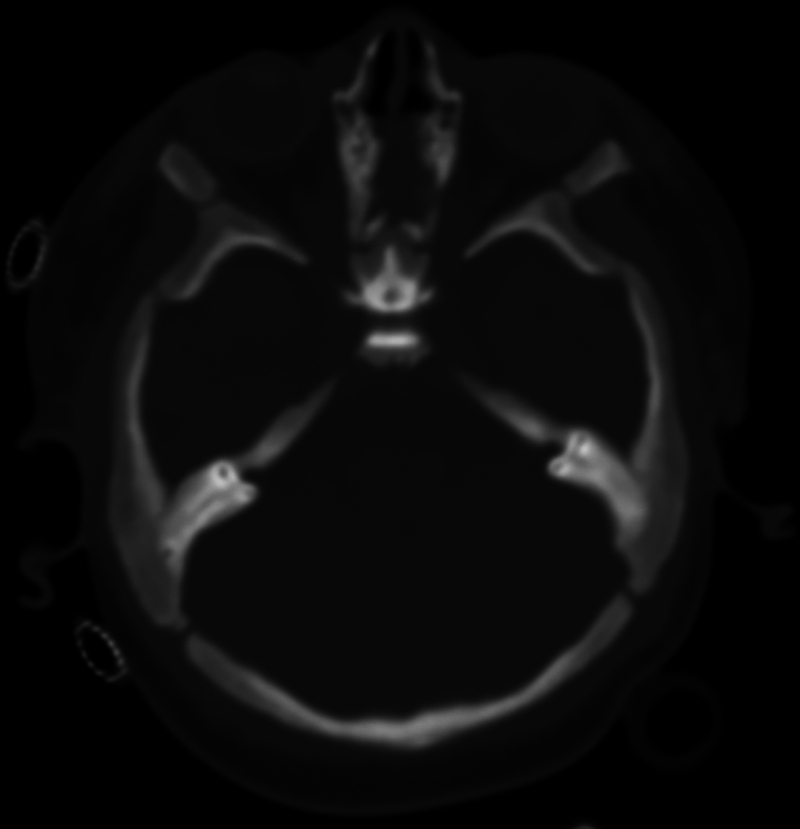
Computed tomography brain showing decompressed optic canal on the right on postoperative day 9.

**Fig. 5:**
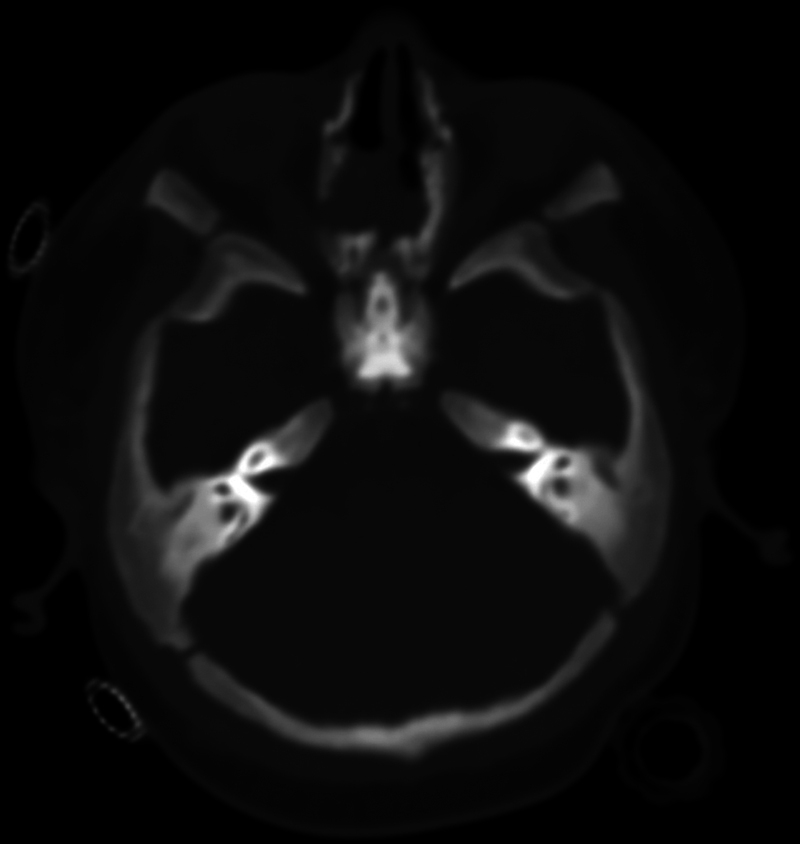
Computed tomography brain showing decompressed optic canal on the left on postoperative day 9.

Postoperatively, the patient was closely monitored in the PICU. The recovery was uneventful, with no postoperative complications. Follow-up assessments revealed significant improvement in the patient's vision, with enhanced visual tracking and responsiveness. On postoperative day 9, computed tomography (CT) brain showed decompression of optic canals, bilaterally ( Figs. 4 , 5 ). At the otorhinolaryngology clinic follow-up on August 5, 2024, the patient showed minimal bleeding and continued improvement in visual function, as evidenced by his ability to follow his mother with his eyes—a notable improvement compared with his preoperative condition. The patient was maintained on saline nasal sprays, with plans for further debridement. Ophthalmology clearance was obtained, and the patient was planned for subsequent BMT.

## Discussion

### Optic Nerve Decompression in Osteopetrosis

Various surgical approaches for optic nerve decompression in osteopetrosis have been explored, each with its own advantages and risks. The historically standard transcranial approach is highly invasive and associated with significant morbidity due to the risks of neurological complications and the extensive nature of the surgery. 6 In contrast, the transorbital approach provides direct access to the orbit through an eyelid or orbital incision, reducing the need for larger incisions but still posing risks to nearby ocular structures and potential visible scarring. 7 The transcaruncular approach is less invasive, accessing the orbit through the caruncle with a hidden incision, minimizing visible scarring and ocular damage but offering less extensive access compared with the transorbital method. Meanwhile, the expanded endoscopic endonasal approach (EEA), as utilized in this case, provides minimal invasiveness, reduced recovery time, and direct access to the optic canal. This approach has evolved considerably with advancements in endoscopic technology, offering a minimally invasive route with fewer complications. 8 9

### Role of Navigation in Endoscopic Surgery

While navigation systems in endoscopic surgery are often considered indispensable, enhancing precision through real-time imaging and guidance, 10 experienced surgeons can achieve desired outcomes without navigation by relying on detailed anatomical knowledge and surgical expertise. This case illustrates that successful bilateral endoscopic endonasal optic nerve decompression can be performed without navigation, offering a cost-effective solution, particularly in resource-limited settings.

### Systematic Review Findings

The systematic review ( Table 1 ) highlights key differences in the age of patients, surgical approaches, and outcomes reported in the literature on optic nerve decompression for osteopetrosis. Notably, most cases involved patients who underwent craniotomy-based approaches such as transcranial or transorbital surgeries, which were historically the standard but are associated with significant morbidity. While a growing number of cases have employed the endoscopic approach, reflecting advancements in surgical techniques and technology, our case stands out as the youngest patient to undergo bilateral endoscopic endonasal optic nerve decompression. Furthermore, unlike other reported endoscopic cases, 11 our procedure was successfully performed without the use of navigation, underscoring that excellent outcomes can still be achieved with surgical expertise, even in resource-limited settings.

**Table 1:** Summary of surgical approaches and outcomes of optic nerve decompression for osteopetrosis cases in the literature

Study	Age, gender	Presentation	Preoperative imaging findings	Navigation (yes/no)	Surgical approach	Complications	Outcome
Moe and Skjaeveland 1969	2 y, F	Decreased visual acuity	Not mentioned	NA	Not mentioned	None	Improved visual acuity
Al-Mefty et al 1988	7 y, M	Progressive visual and hearing loss	Narrowing of optic canals, narrowing of internal auditory meati, generalized sclerosis of skull base	NA	Supraorbital craniotomy	None	Improved visual acuity
	8 y, F	Decreased visual acuity	Severe optic canal narrowing and extensive brain calcification	NA	Supraorbital craniotomy	None	Improved visual acuity and resolution of nystagmus
	17 y, F	Decreased visual acuity	Not mentioned	NA	Supraorbital craniotomy	Not mentioned	Improved visual acuity
	1.5 y, M	Cannot follow objects or walk alone	Not mentioned	NA	Supraorbital craniotomy	Not mentioned	Follows objects, plays with toys, walks alone
	7 y, M	Decreased visual acuity	Not mentioned	NA	Supraorbital craniotomy	Not mentioned	Lost to follow-up
	6 y, M	Decreased visual acuity	Not mentioned	NA	Supraorbital craniotomy	Not mentioned	Improved visual acuity
Haines et al 1988	3.5 y a , M	Developmental delay, decreased visual acuity	Bilateral narrowing of optic foramina	NA	Pterional craniotomy	None	Improved visual acuity
	2 mo a , M	No symptoms	Bilateral narrowing of optic canals	NA	Extradural craniotomy	None	Inadequate decompression, patient died before second attempt
	7 mo a , F	No symptoms	Bilateral narrowing of optic foramina	NA	Pterional craniotomy	None	No improvement, death
	17 mo a , F	Left esotropia	Bilateral narrowing of optic foramina	NA	Pterional craniotomy	None	Improvement of behavior
	8 mo a , M	No symptoms	Optic canals at lower limit of normal	NA	Pterional craniotomy	None	Improved visual acuity, no nystagmus or strabismus
Hwang et al 2000	8 y, F	Decreased visual acuity	Thickened sphenoid bones compressing both optic nerves and superior orbital fissures	NA	Bifrontal craniotomy	None	Improved visual acuity
Medsinge et al 2019	6 mo a , F	Nystagmus, Strabismus	Thickening of the optic strut and narrowing of both optic canals	NA	Endoscopic transcaruncular	None	Improved visual acuity
Yang et al 2024	22 mo, F	Decreased visual acuity	Bilateral narrowing of optic canals	Yes	Endoscopic endonasal	None	Improved visual acuity
	22 mo, F	Nystagmus	Severe compression of the bilateral orbital apices with fluid-filled optic nerve sheaths suggestive of edema	Yes	Endoscopic endonasal	None	Death from other causes
	23 mo, F	Decreased visual acuity	Left-sided optic canal stenosis, left orbital apex narrowing with thinning of the left optic nerve	Yes	Endoscopic endonasal	None	No improvement
Our case	7 mo, M	Not tracking	Small optic canals, suggesting bilateral optic nerve compression	No	Endoscopic endonasal	None	Improved tracking and behavior

Abbreviations: F, female; M, male; NA, not applicable.

a Age listed reflects age at diagnosis, as age at surgery was not reported.

### Significance of Early Intervention

Early intervention is critical for preserving vision and enhancing the quality of life in young patients with optic nerve compression due to osteopetrosis. Multiple case reports in the literature have reported complete visual recovery in infants as young as 6 months of age following early optic nerve decompression secondary to osteopetrosis. 7 8 11 Similarly, Shibata et al documented significant visual improvement in a 12-month-old infant following urgent decompression surgery of a craniopharyngioma. 10 These cases underscore the necessity of early diagnosis and intervention, particularly in young patients. Jenkins et al and Kong et al emphasized the benefits of prompt endoscopic optic canal decompression in pediatric patients aged 5 to 14 years, which reduces morbidity and enhances recovery. 12 13 Genetic testing and imaging are crucial for identifying candidates before irreversible damage occurs. Notably, our 7-month-old patient is among the youngest reported in the literature to undergo this life-changing procedure, underscoring the importance of early surgical intervention in even the youngest patients.

### Cost Considerations

The cost of advanced surgical procedures like endoscopic endonasal optic nerve decompression poses a significant barrier in third-world countries. Limited resources and financial constraints hinder access to specialized treatments. Moe and Skjaeveland emphasized the challenges the need for affordable health care solutions in such settings. 14 Barriers include the high cost of surgical equipment, lack of trained specialists, and inadequate health care infrastructure. Thota et al suggested that international collaborations and training programs for local health care providers could help bridge these gaps. 15 Yang et al demonstrated that specialized care, such as endoscopic endonasal optic nerve decompression, can be delivered successfully in challenging environments with the right support and resources. 11

#### Surgical Pearls and Recommendations

Preoperative planning is essential for optimizing surgical outcomes. Detailed imaging, including CT and MRI, should be obtained to assess the optic canals, orbital anatomy, and bone thickening associated with osteopetrosis, aiding in surgical planning and identifying potential challenges. A multidisciplinary team approach, involving rhinology, neurosurgery, and ophthalmology, is crucial for comprehensive management, particularly in complex cases. Intraoperatively, visualization can be enhanced using a 30-degree endoscope, allowing better access to the medial orbital wall and optic canal. Bone removal should be performed cautiously with powered instruments such as microdebriders and drills, starting with partial middle turbinate resection to improve access. Drilling should cease upon reaching the periorbita to preserve its integrity and prevent excessive pressure on the optic nerve.

To minimize complications, meticulous hemostasis should be achieved using agents such as FloSeal or Surgicel, while careful bone removal near the skull base is necessary to reduce the risk of CSF leaks. Although navigation systems enhance precision, experienced surgeons can achieve excellent outcomes without them by relying on anatomical landmarks and preoperative imaging, particularly in resource-limited settings. Postoperatively, close monitoring is required to detect complications such as infection, scarring, or recurrent optic nerve compression. Regular ophthalmologic assessments should be performed to evaluate visual recovery and identify any emerging complications early. Nasal debridement should also be scheduled to prevent crusting and support proper healing of the nasal cavity.

Patient selection and counseling are integral to optimizing outcomes. Early surgical intervention in pediatric patients is crucial to prevent irreversible vision loss. Parents should be counseled on the risks, benefits, and prognosis to ensure informed decision-making. In cases where navigation or advanced tools are unavailable, reassurance should be provided that favorable outcomes are still possible with an experienced surgical team. 9 15

## Conclusion

This case report highlights the successful management of optic nerve compression in an infant with osteopetrosis using an EEA. Foregoing navigation due to financial constraints did not compromise the precision or outcomes of the surgery, demonstrating that with appropriate surgical expertise, excellent results can be achieved in resource-limited settings. Early diagnosis and intervention were critical in preventing irreversible visual loss and improving the patient's quality of life. This case underscores the importance of minimally invasive techniques in the surgical management of optic nerve compression, particularly in pediatric patients. The successful outcome in this patient, the youngest reported in the literature to undergo such a procedure, emphasizes the benefits of early surgical intervention combined with comprehensive multidisciplinary care.
